# Epidemiological Survey of Hepatitis C Virus Infection in Fife, Scotland

**DOI:** 10.4021/gr2009.10.1316

**Published:** 2009-09-20

**Authors:** Lukman Hakeem, Grace A Thomson, Diptendu N Bhattacharyya

**Affiliations:** a Infectious Diseases Unit, Victoria Hospital (NHS Fife), Kirkcaldy, Fife, United Kingdom, KY2 5AH, UK

**Keywords:** Hepatitis C, Genotype, Injecting drug use, Sustained viral response, Epidemiological survey

## Abstract

**Background:**

HCV infection is of growing public health importance in Scotland. We aim to establish: patient demographics; risk category; year/country of probable infection; referral/follow-up status; and genotypic variance of HCV in Fife.

**Methods:**

Details of all HCV antibody positive patients, referred and assessed at specialist clinics in NHS Fife, until 1st of May 2007 were obtained retrospectively from the Fife hepatitis C database.

**Results:**

In these patients, the ratio of males: female was 2:1, mean age was 36 years, representing a relatively young population, 27.4% of the patients consumed alcohol and 52.4% were smokers. Twelve patients were HIV/HCV co-infected (3.3%). Among the patients, 6.8% had serological evidence of past HBV exposure, 0.5% of patients were HCV/HBV co-infected and 18.8% were vaccinated. Eighty-six percent acquired HCV through injecting drug use and most cases were relatively newly acquired. Referral numbers were on the increase. Thirty-three of patients were under follow-up. Sixty-five percent of patients were genotype 2/3 and 35% were Genotype 1.

**Conclusions:**

Clear patterns were observed in terms of age group, gender, geographical distribution and risk category to facilitate the effective targeting of resources. HCV population in Fife are relatively young, have acquired HCV recently and are mostly of genotype 2/3. This may have a favourable influence on disease progression and cost implications of treating HCV in Fife.

## Introduction

The growing importance of hepatitis C as a public health issue in this country was highlighted in 2000 with the publication of a report by the Scottish needs assessment programme (SNAP). The SNAP report brought together existing initiatives to tackle hepatitis C and made recommendations on how prevention, diagnosis and treatment could be improved. An action plan designed to promote further implementation of SNAP recommendations and also the key messages from the consensus statement, which emerged from the conference in the Royal College of Physicians of Edinburgh in April 2004, was published in June 2005. There were three principal objectives in this action plan. Namely, to reduce the transmission of hepatitis C virus (HCV) among current injecting drug users (IDUs); to diagnose infected persons, particularly those who are most in need of therapy and to provide the optimal care and support for HCV diagnosed persons who are able to benefit [1]. A national clinical guideline (SIGN) for the management of hepatitis C was published in December 2006 due to wide variation that existed across Scotland in the delivery of services to individuals infected with HCV. SIGN guideline provided evidence based recommendations covering all stages of the patient care pathway.

Fife Acute Hospitals (NHS Fife) serve a population of approximately 280,000. In order to evaluate the current hepatitis C case load and management in Fife, we carried out a survey on all patients with a positive HCV antibody test referred and assessed at NHS Fife, until 1st of May 2007. Aims of our survey were to establish the following: (1) Patient demographics of the Fife hepatitis C cohort; (2) Risk category of HCV acquisition; (3) Year of probable infection and country of infection; (4) Referral patterns and current follow-up status of patients; (5) Genotypic variance of chronic hepatitis C patients in Fife.

## Methods

Details of all patients with a positive hepatitis C antibody test, referred and assessed at Fife acute hospitals (NHS Fife), until 1st of May 2007 were obtained retrospectively from the Fife hepatitis C database (part of Scottish Hepatitis C database). However, data on patients who were referred, but have not yet attended for assessment were collected from the referral letter and the laboratory computer records. Data collected include details on patient demographics, details on risk factors for HCV acquisition, source of referral, HCV PCR and genotype results, follow-up status and treatment status. Year of probable infection was defined as the year in which the patient first came into contact with a known HCV risk factor. Continuous variables are presented as mean values and categorical variables in absolute numbers and percentages.

## Results

### Patients demographics of Fife hepatitis C cohort

**Figure 1:**
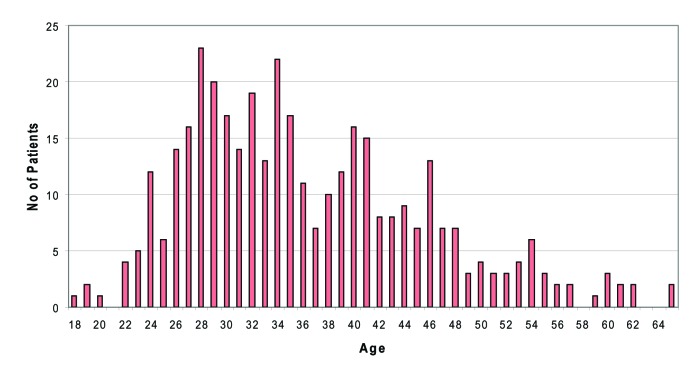
Age distribution of HCV patients (n = 368)

Males out-numbered females by a ratio of 2:1 (246 males, 122 females). Mean age of the patients were 36 years (range 18-65 years). Distribution of cases showed that most patients were in their twenties and thirties, representing a relatively young population. Figure 1 shows the age distribution of the Fife HCV cohort clearly.

Table 1 shows the patient characteristics including ethnicity, smoking/alcohol history, and HIV/ hepatitis B status of Fife HCV cohort.

**Table 1:** Patient demographics of Fife HCV cohort

Characteristic	No. of patients	%
Gender		
Male	246	66.8
Female	122	33.2
Ethnicity		
White	361	98.1
Pakistani	5	1.4
Unknown	2	0.5
Smoking history		
Never	12	3.4
Ex-smoker	9	2.4
Current	193	52.4
Unknown	154	41.8
Previous alcohol history (per week)		
0 units	16	4.3
1 - 21 units	12	3.3
22 - 50 units	5	1.4
> 51 units	44	12.0
Current alcohol intake (per week)		
0 units	46	12.5
1 - 21 units	76	20.7
22 - 50 units	10	2.6
> 51 units	15	4.1
Unknown	144	39.1
HIV status		
Positive	12	3.3
Negative	356	96.7
HBV status		
HepBcAb-, HepBsAB-	100	27.2
HepBcAb-, HepBsAb	69	18.8
HepBcAb+, HepBeAg, HepBsAg-	23	6.3
HepBsAg+	2	0.5
Unknown	174	47.2

### Risk category of HCV acquisition

Our survey showed that 316 of 368 patients (86%) acquired their infection through injecting drug use (IDU), with only a small proportion stating other risk factors. Only 5 patients (1%) were known to have acquired infection through blood/tissue transfer and 5 patients (1%) through heterosexual contact. Risk category was unknown for 42 patients (12%).

### Year of probable infection and country of infection

**Figure 2:**
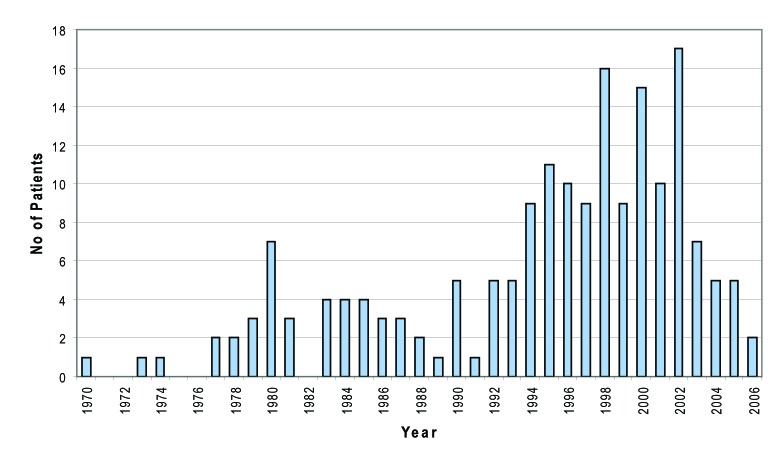
Year of probable infection.

There were very few cases acquired in the seventies, with increasing number of cases throughout the 1980s. However, in the Fife area, most cases of HCV are relatively newly acquired infections, with number of cases increasing throughout the nineties, peaking in 2002. Since 2002, much fewer cases have been acquired (Fig. 2).

Of the 368 patients diagnosed and referred to specialist clinics, 347 patients (94.3%) acquired their infection in Scotland, with further 11 patients (3%) acquiring infection within the rest of UK. Only about 10 patients (2.7%) are known to have been infected out of UK.

### Referral patterns and current follow-up status of patients

**Figure 3:**
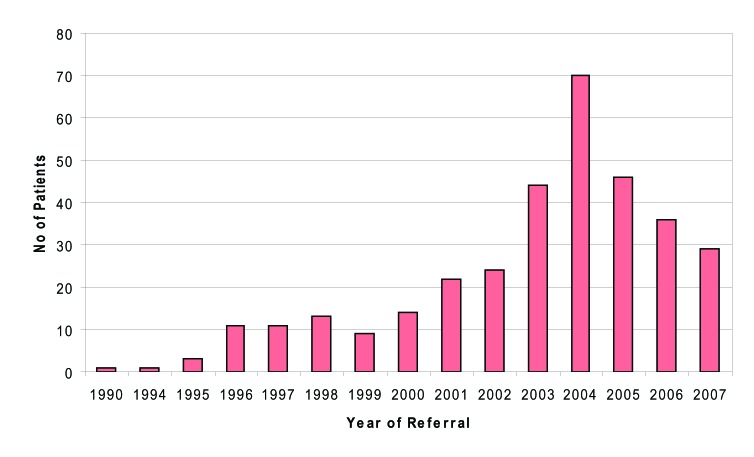
No of patients referred.

Referrals to the specialist clinic were made from variety of different sources. Most referrals, 215 of 368 originated from general practitioners (58%). Sixty-one patients (16.6%) were referred from local genitourinary clinics; Thirty-nine patients (10.6%) from other hospitals or departments; two patients (0.5%) from mental health services; Twelve patients (3.3%) from the obstetrics unit; one patient (0.3%) from blood transfusion service; two patients (0.5%) from prison services and 5 (1.4%) from alcohol and addiction services. Thirty-one patients (8.4%) were self-referrals. In this decade, the number of patients referred annually has been on the increase, with current 5 month figures for 2007 similar to those numbers referred in the previous 12-month period. (Fig. 3).

**Figure 4:**
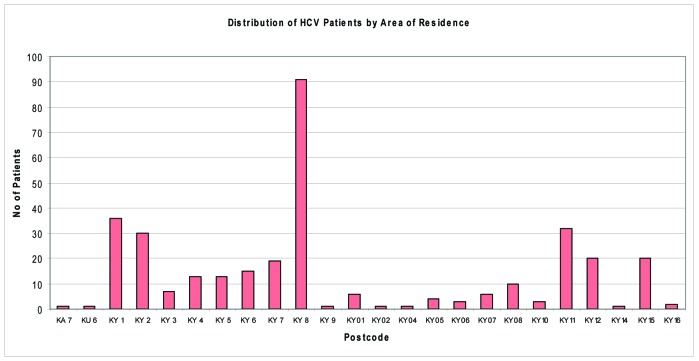
Number of cases by area of residence.

Examining patient’s area of residence and general practitioner, the geographical distribution of patients could be examined, and resources could be targeted accordingly. Figure 4 shows that a large proportion of cases reside in Kirkcaldy (KY1, KY2) and Methil /Leven (KY8) areas. Victoria Road surgery, Kirkcaldy Health Centre and Muiredge surgeries represent most of the Fife HCV cohort of patients.

Due to the chaotic lifestyle of most HCV-infected individuals, it is not unexpected that a large proportion of patients do not attend hospital follow up. In the Kirkcaldy patient cohort, 83 patients (22.6%) have never attended despite repeated appointments being offered, with a further 148 patients (40%) had only attended occasionally, but not recently. General Practitioners are normally informed by writing to re-refer these patients if appropriate, after they had failed to attend at least three clinic appointments. Only 121(33%) patients referred to specialist clinics are currently under follow up for their disease. Thirteen patients (3.6%) did not require follow up due to persistently negative Hepatitis PCR results and three patients (0.8%) had died.

Currently only 10 patients in the Fife cohort are known to be cirrhotic (3.5 % of patients assessed in hospital). To date no patients are known to have developed hepatocellular carcinoma.

### The genotypic variance of chronic hepatitis C patients in Fife

Hepatitis C genotype has implications on treatment response rate and duration. Genotype information was available for 112 patients. Genotype 1 accounted for 39 patients (35%). Six patients (5%) were found to be Genotypes 2 and 67 patients (60%) were genotype 3.

## Discussion

It is estimated that around 200 million people worldwide are infected with the Hepatitis C virus (HCV) [1]. The virus was identified in 1989, and an antibody test to detect its current or past presence became available in 1991 [1, 2]. HCV is transmitted primarily through percutaneous exposure though it can be spread by unprotected sexual intercourse and from mother to child during pregnancy or at the time of birth [1, 3]. In resource poor countries, HCV is mainly transmitted through the receipt of infected blood or blood products and through the re-use of unsterile needles and syringes for health care purposes. Between 5-15% of chronically infected persons develop cirrhosis within 20 years of infection [2, 4].

Factors associated with more rapid disease progression are older age at time of infection, male gender, excessive alcohol consumption and co-infection with HIV [5, 6].

SIGN guideline recommends that when estimating the likely rate of progression of liver disease, age at infection, gender and ethnicity should be considered [7]. Our survey showed that HCV infections in males were far more common than in females (ratio 2:1) in Fife. Age distribution in the Fife cohort showed that most patients were in their twenties and thirties (mean age 36 years). Fife cohort thus represents a relatively young population, when compared to the Edinburgh patient cohort where most patients are between the ages of 40 and 59 years (Edinburgh Royal Database). This factor may have a favourable influence on the rate of disease progression as increasing age at time of infection is associated with more rapid progression of liver fibrosis and reduced time from infection to cirrhosis. Age over 40 years at the time of infection is particularly associated with more rapid progression [8, 9]. Variations in disease progression have been observed in patients of different race. Cohort studies have demonstrated that disease progressed less rapidly in African-American than non African-American patients [10, 11]. However a significant majority of patients in Fife (98.1%) are of white ethnicity and therefore ethnicity may not be a significant factor in estimating disease progression in Fife.

Heavy alcohol consumption in patients with chronic hepatitis C (CHC) is associated with more severe liver disease including cirrhosis, end stage liver disease and hepatocellular cancer [12, 13]. Even moderate amounts of alcohol (within government recommended guidelines) have been associated with increased liver fibrosis compared to those who abstain [8, 12]. Patient demographics in Fife shows that 27.4% patients referred to our unit consumed alcohol at the time of consultation, 20.7 % in moderation (up to 21 units per week), 6.7% in excess (above 22 units per week). Alcohol history was unknown for 39.1% of patients. This may be partly related to some patients not attending the clinic despite being referred and the alcohol history not being available on the referral letter. Past alcohol history showed that at least 16.7% consumed alcohol in the past of which 13.4% consumed alcohol in excess. Patients with CHC should be advised that drinking alcohol, even in moderation can accelerate progression of liver disease [7].

Smoking is an independent risk factor for the progression of hepatic inflammation and fibrosis in patients with CHC [14, 15], although no data were identified on the impact of stopping smoking. Nevertheless, SIGN guidelines recommends that patients with CHC should be advised that smoking tobacco can accelerate progression of liver disease. Among these patients, 52.4% referred for assessment of HCV infection were smokers. Smoking history was unknown in 41.8% of cases. It is therefore vital that a proper smoking history is obtained when the patient attends the general practitioner initially or the specialist clinic subsequently, in order to advise accordingly.

There is an increased rate of progression to end-stage liver disease in patients with HIV and HCV co-infection compared to those with HCV mono-infection [16]. With the availability of highly active antiretroviral therapy (HAART) for HIV management, end stage liver disease has become the leading cause of hospitalisation and death in this group. There are only 12 HIV/HCV co-infected patients in Fife (3.3%). The increased rate of progression to decompensated liver disease in these patients should prompt early consideration of antiviral therapy. Patients who are infected with HCV who have serological evidence of current or past infection with hepatitis B virus (HBV) are more likely to have advanced liver disease [17, 18], and it is important to consider active or previous infection with HBV when estimating the rate of progression of liver disease. Our survey showed that 6.8% of patients had been exposed to HBV in the past of which 0.5% is currently co-infected. HBV status is unknown for 47.2% of the Fife cohort. SIGN guidelines recommend considering vaccination against hepatitis A and B in patients infected with HCV. A consensus report on the treatment of HCV recommends vaccination for hepatitis B but not hepatitis A [7, 17]. To the best our knowledge only 18.8% of patients in Fife cohort were vaccinated for HBV (HepBcAb-, HepBsAb+). This may be partly due to the fact that the HBV status is unknown in a significant proportion of patients and therefore more patients may have been vaccinated in the past. It is therefore vital that all patients are tested for HIV and HBV status including current/past infection and immunity status to HBV, and offered vaccination accordingly.

HCV has been circulating among injecting drug users in Scotland since at least the mid 1970s. By 2004, it was estimated that 50,000 persons were living with HCV infection in Scotland (1% of Scotland’s population). Of the 50,000 infected persons, 37,500 (75%) were estimated to be chronically infected (12,400 diagnosed and 25,100 undiagnosed) and, thus, at the risk of developing cirrhosis [2]. A separate modelling exercise estimated that 33,000 (88%) of all those chronically infected were IDUs (24800 former and 8200 current IDUs) [19].

Our survey showed that 86% of all patients in Fife infected with HCV acquired their infection through injecting drug use, with only a small proportion stating other risk factors. Only 1% of patients were known to have acquired infection through blood/tissue transfer, and this may reflect the fact that they have yet to be identified, or were possibly identified through the Blood Transfusion Service look back scheme in 1995, and were referred to other health board areas.

In Fife area, most of cases of HCV are relatively newly acquired infections, with number case increasing throughout the nineties, peaking in 2002 and declining since then. This data may reflect an actual decline in infections, or these patients may simply not have been identified or referred on to specialist care at this time. However, it is interesting to note that the new tiered needle exchange system was initiated in 2002 in Fife and this may be partly responsible for the above figures. Data available thus far suggests that the HCV epidemic in Fife followed on from that in Edinburgh, where infections increased steadily throughout the early eighties and then the number of cases tailed off in to the nineties. Also a majority (94%) of patients referred to our unit acquired their infection in Scotland with only a small number (2.7%) known to have been infected abroad.

Referral to specialist care should be considered for all patients with active HCV infection (HCV RNA positive) and not restricted to potential candidates for antiviral therapy. Reasons for the great majority of HCV diagnoses not having entered specialist care include a PCR negative test result indicating clearance of infection, failure to attend following referral, failure to be referred and, probably most commonly, continuing injecting drug use, rendering individuals ineligible for therapy [2]. Specialist clinics are often a source of information for patients and relatives, including health promotion and methods of avoiding secondary transmission of the virus. Annual number of referrals has been on the increase in Fife this decade. SIGN guideline recommends that individuals, including injecting drug users, diagnosed with chronic HCV should be offered integrated multidisciplinary care as it can maximise their uptake of, and retention in services. Follow up status in Fife shows that only 33% of patients are currently under follow-up. A large proportion of patients (22.6%) never attended the specialist clinics despite being referred. Further 40% were lost to follow up. This group of patients need to be targeted for re-engagement with services to address their HCV infection and resources could be targeted according to the geographical distribution of patients in Fife.

HCV genotype information was only available for 30.4% of patients. This is most likely due to the high proportion of patients not attending the clinic, were lost to follow up or genotype only being tested when patients were considered suitable for treatment. Also routine genotype testing was not available until recently. Of the patients who were tested for genotype in Fife the majority (65%) were found to be genotype 2 and 3. This may have important cost implications as the optimal duration of treatment for patients with genotype 2 or 3 is 24 weeks, compared to patients with genotype 1 or 4, where the optimal duration is 48 weeks [20-22]. Furthermore, the chances of achieving sustained viral response, which should be used as a marker for viral clearance [7], is greater for genotype 2 and 3 (73-82%) compared to genotype 1 disease (41-51%) [23, 24].

Sustained viral response (SVR) has become the accepted objective of treatment programmes for CHC (negative HCV RNA 6 months after completion of treatment). Viral relapse is uncommon, mortality and risks of developing cirrhosis and primary hepatocellular carcinoma is reduced after SVR [7, 25, 26]. Treatment should be considered for patients with mild/moderate CHC, patients with CHC with normal ALT levels, patients with HIV co-infection, Hepatitis B co-infection and patients on stable drug treatment programmes. Also patients with compensated cirrhosis should be considered for therapy, unless contraindicated [7].

Only 3.5% assessed in our specialist clinic are known to be cirrhotic. This low proportion is most likely to reflect the length of time patients have been infected with HCV, and can be expected to increase in the future. Assuming the continuation of current rates of antiviral therapy administration, the number of HCV-infected IDUs developing decompensated cirrhosis in Scotland is estimated to approximately double from 80 in 2000 to 150 in 2020 [2, 8]. If the relatively low current levels of antiviral therapy do not increase in the future, the numbers of HCV infected persons with severe disease will increase considerably. Reducing the burden of such disease over the next two decades involves increasing the numbers of chronically HCV infected persons treated, but also ensuring that those treated are the ones most at risk of progressing to cirrhosis, liver failure and liver cancer.

In conclusion, patient demographics in Fife show clear patterns with regards to the nature of the patients in terms of age group, gender, lifestyle, risk category and geographical distribution that will facilitate the effective targeting of resources. Majority of HCV patients in Fife are IDUs, are relatively young and have acquired HCV recently. This may influence disease progression favourably, as increasing age at time of infection is associated with more rapid progression of HCV infection. This finding gives us a great opportunity to treat more individuals, achieving SVR and therefore reducing mortality, risks of developing cirrhosis and hepatocellular carcinoma. Significant majority of patients in Fife are of genotype 2 and 3 which requires a shorter duration of treatment and has a better outcome thus having a favourable influence on cost implications of treating HCV.
